# APOE2 Is Associated with Spatial Navigational Strategies and Increased Gray Matter in the Hippocampus

**DOI:** 10.3389/fnhum.2016.00349

**Published:** 2016-07-13

**Authors:** Kyoko Konishi, Venkat Bhat, Harrison Banner, Judes Poirier, Ridha Joober, Véronique D. Bohbot

**Affiliations:** Department of Psychiatry, Douglas Mental Health University Institute, McGill UniversityVerdun, QC, Canada

**Keywords:** hippocampus, spatial memory, navigation, APOE

## Abstract

The Apolipoprotein E (APOE) gene has a strong association with Alzheimer’s disease (AD). The ε4 allele is a well-documented genetic risk factor of AD. In contrast, the ε2 allele of the APOE gene is known to be protective against AD. Much of the focus on the APOE gene has been on the ε4 allele in both young and older adults and few studies have looked into the cognitive and brain structure correlates of the ε2 allele, especially in young adults. In the current study, we investigated the relationship between APOE genotype, navigation behavior, and hippocampal gray matter in healthy young adults. One-hundred and twenty-four healthy young adults were genotyped and tested on the 4on8 virtual maze, a task that allows for the assessment of navigation strategy. The task assesses the spontaneous use of either a hippocampus-dependent spatial strategy or a caudate nucleus-dependent response strategy. Of the 124 participants, 37 underwent structural magnetic resonance imaging (MRI). We found that ε2 carriers use a hippocampus-dependent spatial strategy to a greater extent than ε3 homozygous individuals and ε4 carriers. We also found that APOE ε2 allele carriers have more gray matter in the hippocampus compared to ε3 homozygous individuals and ε4 carriers. Our findings suggest that the protective effects of the ε2 allele may, in part, be expressed through increased hippocampus gray matter and increased use of hippocampus-dependent spatial strategies. The current article demonstrates the relationship between brain structure, navigation behavior, and *APOE* genotypes in healthy young adults.

## Introduction

Apolipoprotein E (APOE) is a well-documented risk gene for Alzheimer’s disease (AD; Corder et al., 1993). The association between the ε4 allele and cognitive performance has been well characterized in all age groups (Yu et al., 2000; Small et al., 2004; Alexander et al., 2007; Mondadori et al., 2007; Acevedo et al., 2010; Bunce et al., 2011). However, few studies have looked at the protective effects of the ε2 allele in older adults, and even fewer have looked at the cognitive and structural correlates of this allele in young adults. An important reason for the scarce body of literature comparing ε2 carriers and non-carriers may be the lack of sensitivity of commonly used standard neuropsychological tests. Alexopoulos et al. (2011) and Alexander et al. (2007) tested young adults on standard memory and executive function tasks and both studies found no differences in performance. In older adults, the ε2 allele has been shown to be associated with reduced risk and delayed onset of AD (Corder et al., 1994; Polvikoski et al., 1995; Farrer et al., 1997; Lippa et al., 1997; Tiraboschi et al., 2004; Conejero-Goldberg et al., 2014; Serrano-Pozo et al., 2015), lower levels of neuritic plaque formation (Corder et al., 1994; Tiraboschi et al., 2004), and slower rates of hippocampal atrophy (Chiang et al., 2010). It is also associated with longevity (Schachter et al., 1994; Blanche et al., 2001; Frisoni et al., 2001) and reduced cognitive decline with age (Helkala et al., 1995; Hyman et al., 1996; Wilson et al., 2002). In children, the ε2 allele is associated with greater cortical thickness in the entorhinal cortex (Shaw et al., 2007), a structure that provides major inputs to the hippocampus. Similarly, in young adults, ε2 allele carriers have been shown to have a larger hippocampus compared to ε4 allele carriers (Alexopoulos et al., 2011). However, despite the evidence of structural differences in ε2 allele carriers from an early age, to date, no studies have found any cognitive correlates that are sensitive to these structural differences in young adults.

Spatial memory is a function that critically depends on the hippocampus (O’Keefe and Dostrovsky, 1971) and spatial memory impairments are often one of the first symptoms observed in AD (Klein et al., 1999; Mapstone et al., 2003; Hort et al., 2007). This suggests that spatial memory tasks may be particularly sensitive in detecting early changes in cognitive function associated with hippocampal atrophy. Iaria et al. (2003) demonstrated that multiple strategies can be used to navigate in an environment. These strategies rely on different brain networks, one of which involves the hippocampus. Iaria et al. (2003) found that approximately 50% of young adults spontaneously use a spatial strategy while the other 50% spontaneously use a response strategy. The spatial strategy involves building relationships between landmarks in the environment to form a cognitive map (O’Keefe and Nadel, 1978). This form of navigation critically depends on the hippocampus and patients with hippocampal lesions are impaired at this form of navigation (Bohbot et al., 2004). In contrast, the response strategy involves learning stimulus-response associations, such as a series of right and left turns from specific points in space (McDonald and White, 1994; Packard and Mcgaugh, 1996). This strategy critically depends on the caudate nucleus of the striatum (McDonald and White, 1994; Packard and Mcgaugh, 1996). This form of navigation does not depend on the hippocampus and patients with hippocampal lesions perform similar to normal controls when using a response strategy (Bohbot et al., 2004). Young adults who spontaneously use a spatial strategy have more functional magnetic resonance imaging (fMRI) activity and gray matter in the hippocampus (Iaria et al., 2003; Bohbot et al., 2007). In contrast, young adults who spontaneously use a response strategy have more fMRI activity and gray matter in the caudate nucleus. It was further demonstrated that there is an inverse relationship between the two memory systems such that more gray matter in the caudate nucleus is associated with less gray matter in the hippocampus and vice versa (Bohbot et al., 2007, 2013). Therefore, navigation strategies are sensitive to the predominant use of and gray matter in the hippocampus and caudate nucleus memory systems. As such, navigation strategies is a sensitive measure to structural differences in the hippocampus in young adults and may be an ideal way to detect cognitive differences in ε2 allele carriers.

We hypothesized that in young adults, APOE ε2 allele carriers will use spatial strategies to a greater extent and have more gray matter in the hippocampus in contrast to ε3 homozygous individuals and ε4 allele carriers. Given that a larger hippocampus is associated with decreased cognitive impairment and decreased risk of AD, understanding the relationship between the ε2 allele and the hippocampus may help us better understand the mechanism behind the protective effects of the ε2 allele.

## Materials and Methods

### Participants

One-hundred and twenty-four healthy young adult participants aged 18–37 took part in the behavioral study at the Douglas Mental Health University Institute. All participants were right-handed as determined by a phone screening questionnaire that assesses handedness. Of the 124, 37 participants underwent structural MRI. Participants were assessed based on a phone screening questionnaire and exclusion criteria. Participants had no personal history of neurological or psychiatric disorders and no history of alcohol or drug abuse. The participants and experimenters were blind to the genotype status. The study received ethical approval from the institutional review boards at the Douglas Mental Health University Institute and the Montreal Neurological Institute at McGill University. Informed consent was obtained from all participants in accordance with the guidelines of the institutional review boards at the Douglas Mental Health University Institute and the Montreal Neurological Institute at McGill University.

### Behavioral Testing

**Figure 1:**
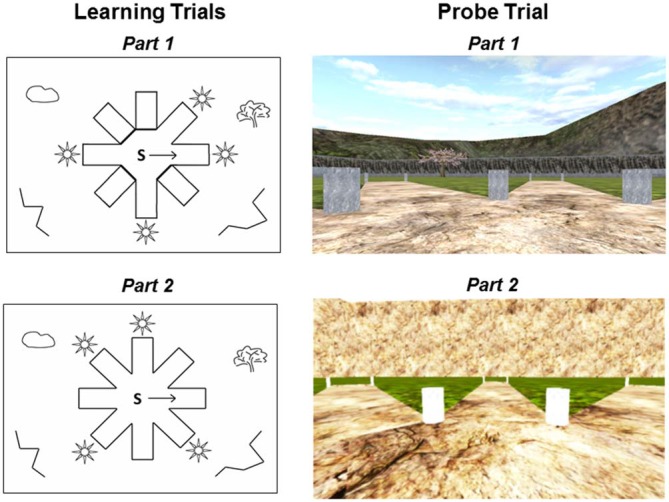
**Schematic drawings and first person views of the 4-on-8 virtual maze.** Left: Top-down schematic drawing of the learning trials. During part 1 of the learning trials (top left), four pathways are open and four pathways are closed. “S” depicts the starting position and the arrow indicates the direction that the participant is facing when first placed in the environment. In part 2 of the learning trials (bottom left), all pathways are open and there are no barriers. Right: First-person view of the probe trial. During part 1 of the probe trial (top right), all landmarks are visible in the environment. Part 1 of the probe trials is the same as part 1 of the learning trials. During part 2 of the probe trial (bottom right), a wall is erected around the maze and all landmarks are removed from the environment.

Participants were tested on the 4/8VM (Iaria et al., 2003; Bohbot et al., 2004, 2007), a computer-based virtual reality navigation task modeled after a maze traditionally used in rats (Harley, 1979; Walker and Olton, 1979). The 4/8VM is an eight-arm radial maze with a central starting location, surrounded by distinct landmarks (two trees, a sunset, and mountains; Figure 1). In the 4/8VM, each participant performs five trials. Each trial is comprised of two parts. In part 1, there are four open and four blocked pathways. Participants are asked to go down the four open pathways to retrieve objects located in a pit at the end of the pathway. After all four objects are picked up, participants are presented with part 2 of the task. In part 2, all eight pathways are open and participants are instructed to avoid the pathways they previously visited in order to retrieve the target objects. During the first three trials, participants are able to solve the task by using either a spatial or response strategy. The spatial strategy involves building a cognitive map of the environment based on relationships between objects in the environment and the rewarded pathways. The response strategy involves memorizing a series of pathways to be visited using a pattern or numbering system, for example, “going clockwise, take the first left after leaving the start position, then skip one pathway and take the next two pathways”. After three trials, if participants reach the learning criterion (retrieve all four objects in part 2 of any trial with zero errors), they are presented with a probe trial. In the probe trial, part 1 is the same as part 1 in the three previous trials. However, in part 2 of the probe trial, a wall is erected around the radial maze, blocking the participants’ view of the environmental landmarks. Participants are asked to again retrieve the four objects by avoiding previously visited pathways. After the probe, participants are administered another trial similar to the first three trials. After completion of the task, participants are administered a verbal report and asked to describe their approach to navigating the environment. Based on the verbal report, participants are classified as spatial learners if they report using at least two landmarks to remember the target locations and do not mention using a pattern or sequence of open and closed pathways. Participants are classified as response learners if they report using a pattern or sequence of open and closed pathways to remember the target locations (Iaria et al., 2003; Bohbot et al., 2007). The probe trial is designed to support the strategy classification obtained from verbal reports. In previous studies, spatial learners who relied on landmarks were impaired on the probe trial while response learners who used a sequence or pattern made few errors, showing that their performance is undisturbed by the removal of landmarks (Iaria et al., 2003; Bohbot et al., 2007). Consequently, errors on the probe trial are indicative of the fact that people relied on landmarks when navigating in the environment.

Participants were also tested on the Rey Auditory Verbal Learning Test (RAVLT) in order to assess verbal memory (Rey, 1964). During the RAVLT, participants receive five trials to learn a list of 15 words and they are asked to recall them after an interference trial and after a delay of 30 min. The sensitivity of the 4/8VM can be gauged by comparing the results with those obtained from standard neuropsychological tests such as the RAVLT.

### Genotyping

Blood samples were collected at the Douglas Mental Health University Institute. DNA extraction followed by PCR amplification and *APOE* genotyping was completed after behavioral testing. The *APOE* genotyping was done at Genome Quebec with TaqMan chemistry (Applied Biosystems). None of the participants were excluded from the genotyping. Genotype frequencies for rs429358 (*p* = 0.42) and rs7412 (*p* = 0.91) were in line with the Hardy-Weinberg equilibrium.

### Magnetic Resonance Imaging (MRI) Protocol

Structural MRI scans were obtained at the Montreal Neurological Institute using a 1.5 T Siemens Sonata MRI scanner. Participants were comfortably placed in the scanner with their heads immobilized using an air cushion. An anatomical scan of approximately 15 min was taken for each participant. A 3D gradient echo acquisition was used to collect 160 contiguous 1 mm isometric T1-weighted images in the sagittal plane (*TR* = 22, *TE* = 10, flip angle = 30°, 140 1 mm sagittal slices). The rectangular field of view (FOV) for the sagittal images was 256 mm (SI) by 224 mm (AP).

### Behavioral Statistical Analysis

Participants were divided into three groups based on *APOE* genotypes: *ε*2 carriers (*n* = 18), ε3/ε3 individuals (*n* = 72) and ε4 carriers (*n* = 30). There was one participant with ε2/ε2 who was included in the *ε*2 carrier group and one ε4/ε4 who was included in the ε4 carrier group. There were four participants with ε2/ε4 alleles and they were excluded from the study.

All statistical analyses were performed using SPSS (version 22). Where applicable, bootstrapped bias-corrected and accelerated 95% CIs were used to assess statistical significance and account for deviations from parametric assumptions.

The frequencies of participants using the two strategies were contrasted between the three *APOE* genotype groups using a chi-squared analysis. *Post hoc* analyses were performed to compare strategy proportion differences between ε2 carriers and ε4 carriers, ε2 carriers and ε3/ε3 individuals, and ε4 carriers and ε3/ε3 individuals.

Performance on the RAVLT as well as performance on the 4/8VM probe trial was compared between spatial and response learners and between the three *APOE* genotype groups using ANOVAs. Sex, age, and education were included as covariates for these analyses.

In order to assess whether navigation strategies are more strongly associated with *APOE* genotypes than performance on the RAVLT, we performed a multinomial logistic regression with genotypes as the dependent variable. The goal of the multinomial logistic regression was not to build a suitable predictive model but rather to interpret the coefficients in order to examine the relationship between the experimental variables (navigation strategy and RAVLT performance) and the dependent variable (APOE genotype). The ε2 group was indicated as the reference category. Strategy was inserted as a fixed factor (response strategy = 0, spatial strategy = 1), and RAVLT total recall, after-interference, and delayed recall scores were inserted as continuous variables. Sex, age, and education were also included as covariates.

### MRI Statistical Analysis

Voxel-based morphometry (VBM) was used to analyze the structural MRI scans. VBM is a computational approach to neuroanatomy that measures differences in local density or shape of brain tissue, through a voxel-wise comparison of structural MRI images. This method allows for an analysis of the whole brain. MRI scans were first corrected for intensity non-uniformity (shading artifact) using the N3 software package (Sled et al., 1998). Scans were then spatially normalized by linear transformation into a standard stereotaxic Talairach space using the MNI305 atlas (Talairach and Tournoux, 1988; Collins et al., 1994). Each voxel was automatically labeled as white matter, gray matter, cerebrospinal fluid, or background using Intensity Normalized Stereotaxic Environment for the Classification of Tissues (INSECT; Zijdenbos et al., 2002). The classification of each voxel is based on the voxel intensity and spatial probability. A mask of the skull and dura were then created for each participant and used to remove the skull and dura from the images. The gray matter was smoothed using a 6 mm full-width at half-maximum (FWHM) Gaussian kernel. Participants were divided into three groups based on *APOE* genotypes: *ε*2 carriers (*n* = 5), ε3/ε3 (*n* = 21) and ε4 carriers (*n* = 11), and the genotype groups were contrasted against each other. Based on our* a priori* hypothesis (Iaria et al., 2003; Bohbot et al., 2007), regions of interest analysis was performed in the hippocampus and an uncorrected *p*-value threshold of *p* < 0.001 was used (*N* = 16: *t* = 3.7; *N* = 26: *t* = 3.5; *N* = 37: *t* = 3.3). The borders of the hippocampus were objectively determined by sulci and gyri previously defined (Bohbot et al., 1998). For the whole brain, Bonferroni correction for multiple comparisons was used to calculate the *t*-statistical threshold (*N* = 16: *t* = 8.22 at *p* < 0.05; *N* = 26, *t* = 6.3 at *p* < 0.05; *N* = 37: *t* = 5.70 at *p* < 0.05). Sex, age, education, and navigation strategy were included as co-variates in the analyses.

## Results

We recruited 124 healthy young participants, all of which were genotyped and tested on the 4/8VM. Four of the 124 participants carried the ε2/ε4 genotype and were excluded from the study. As such, a total of 120 healthy young adults completed the 4/8VM and RAVLT (Mean age = 23.83 ± 4.32, education = 15.65 ± 2.10, 64 men, 56 women). Thirty-seven of the 120 participants (Mean age = 25.00 ± 4.26, education = 15.84 ± 1.88, 19 men, 18 women), underwent a structural MRI scan (Table 1). There was no difference in demographics between the complete sample of 120 participants and the 37 participants that underwent the MRI scan (Table 1). No differences were found in the demographic characteristics among different *APOE* groups in both the whole group and within the individuals who underwent an MRI scan (Table 2).

**Table 1:** **Demographic characteristics of participants in the behavioral sample and magnetic resonance imaging (MRI) sub-sample**.

	**Behavioral sample**	**MRI sub-sample**	**Statistics**
N	120	37	
Strategy	Spatial = 39; Response = 81	Spatial = 16; Response = 21	*χ*^2^ = 1.43; *p* = 0.23
APOE	ε2 = 18; ε3/ε3 = 72; ε4 = 30	ε2 = 5; ε3/ε3 = 21; ε4 = 11	*χ*^2^ = 0.34; *p* = 0.85
Age	23.83 (±4.32)	25.00 (±4.26)	*p* = 0.15; Bootstrap Bca 95%CI [−2.79,0.33]
Sex	*F* = 56; *M* = 64	*F* = 18; *M* = 19	*χ*^2^ = 0.05; *p* = 0.83
Education	15.65 (±2.10)	15.84 (±1.88)	*p* = 0.59; Bootstrap Bca 95%CI
RAVLT:			
Total Recall	58.53 (±7.00)	59.08 (±6.29)	*p* = 0.64; Bootstrap Bca 95%CI [−3.05,2.18]
After interference	12.43 (±2.46)	12.62 (±2.14)	*p* = 0.66; Bootstrap Bca 95%CI [−1.04,0.67]
Delayed recall	12.45 (±2.20)	12.68 (±1.99)	*p* = 0.54; Bootstrap Bca 95%CI [−0.94,0.55]

**Table 2:** **Demographic characteristics of Apolipoprotein E (APOE) groups in the behavioral sample and MRI sub-sample**.

				Statistics		
	ε2 carriers	ε3/ε3	ε4 carriers	ε2 carriers vs. ε3/ε3	ε3/ε3 vs. ε4 carriers	ε2 carriers vs. ε4 carriers
**Behavioral sample**
N	18	72	30
Age	23.06 (±4.12)	24.34 (±4.59)	23.07 (±3.68)	*p* = 0.49; Bootstrap Bca 95%CI [−3.13,1.02]	*p* = 0.36; Bootstrap Bca 95%CI [−0.34,2.81]	*p* = 1.00; Bootstrap Bca 95%CI[−1.97,2.30]
Sex	*F* = 11; *M* = 7	*F* = 33; *M* = 39	*F* = 12; *M* = 18		*χ*^2^ = 2.07; *p* = 0.36	
Education	15.33 (±1.41)	15.88 (±2.26)	15.30 (±2.02)	*p* = 0.59; Bootstrap Bca 95%CI [−1.38,0.23]	*p* = 0.42; Bootstrap Bca 95%CI [−0.32,1.40	*p* = 1.00; Bootstrap Bca 95%CI [−0.94,0.89]
**MRI sub-sample**
N	5	21	11
Age	23.20 (±2.49)	25.14 (±4.52)	25.55 (±4.48)	*p* = 0.64; Bootstrap Bca 95%CI [−4.82,0.97]	*p* = 0.97; Bootstrap Bca 95%CI [−3.88,2.86]	*p* = 0.58; Bootstrap Bca 95%CI [−5.87,1.00]
Sex	*F* = 4; *M* = 1	*F* = 10; *M* = 11	*F* = 4; *M* = 7		*χ*^2^ = 2.64; *p* = 0.27	
Education	16.40 (±0.89)	16.14 (±1.98)	15.00 (±1.84)	*p* = 0.96; Bootstrap Bca 95%CI [−0.81,1.39]	*p* = 0.23; Bootstrap Bca 95%CI [−0.30,2.50]	*p* = 0.35; Bootstrap Bca 95%CI [−0.11,2.80]

### APOE and Navigation Strategies

**Figure 2:**
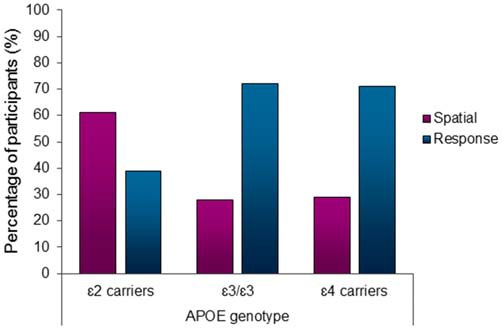
**Apolipoprotein E (APOE) ε2 carriers used the hippocampus-dependent spatial strategy significantly more than the other genotype groups (*χ*^2^ = 7.91, *df* = 2, *p* < 0.05).** 61% of APOE ε2 carriers used a spatial strategy in contrast to only 28% of ε3/ε3 individuals and 29% of ε4 carriers using a spatial strategy.

There was a significantly higher proportion of spatial learners in the ε2 carrier group compared to ε3/ε3 and ε4 carrier genotype groups (*χ*^2^ = 7.91, *df* = 2, *p* = 0.02; Figure 2). The standardized residuals suggest that the significant difference is driven by the ε2 carriers (spatial learners standard residuals: ε2 = 2.1, ε3 = −0.7, ε4 = −0.6). *Post hoc* comparisons revealed that ε2 carriers use spatial strategies significantly more than both ε3/ε3 individuals (*χ*^2^ = 7.09, *df* = 1, *p* = 0.01) and ε4 carriers (*χ*^2^ = 5.58, *df* = 1, *p* = 0.02). There is no difference in the proportion of spatial and response learners between the ε3/ε3 individuals and ε4 carriers (*χ*^2^ = 0.01, *df* = 1, *p* = 0.91). Sixty-one percent of *APOE* ε2 carriers spontaneously used a spatial strategy in contrast to ε3/ε3 (28%) individuals and ε4 (29%) carriers, who showed a significantly lower probability of using spontaneous spatial strategies in favor of response strategies (Figure 2).

Spatial learners made more errors on the probe trial than response learners (*F*_(1,119)_ = 3.63; *p* = 0.06; bootstrapped BCa 95%CI [−0.36,−0.004]), confirming that they relied on landmarks to a greater extent to find the target objects. Spatial and response learners did not differ in performance on the RAVLT (overall *F*_(3,113)_ = 1.37, *p* = 0.26; total recall: *F*_(1,119)_ = 2.92, *p =* 0.09, bootstrapped BCa 95%CI [−5.24,0.81]; after-interference: *F*_(1,119)_ = 2.93, *p* = 0.09, bootstrapped BCa 95%CI [−1.84,0.24]; delayed recall: *F*_(1,119)_ = 1.40, *p* = 0.24, bootstrapped BCa 95%CI [−1.36,0.35]).

The *APOE* genotype groups did not differ in performance on the probe trial of the 4/8VM (*F*_(2.119)_ = 0.25, *p* = 0.78). *Post hoc* analyses indicate that there was no difference between ε2 carriers and ε3/ε3 individuals (*p* = 0.61; bootstrapped BCa 95%CI [−0.28,0.17]), ε2 carriers and ε4 carriers (*p* = 0.56; bootstrapped BCa 95%CI [−0.34,0.19]), and ε3/ε3 individuals and ε4 carriers (*p* = 0.61; bootstrapped BCa 95%CI [−0.26,0.13]). On the RAVLT, ε2 carriers, ε3/ε3 individuals, and ε4 carriers had equal performance on total recall (*F*_(2.119)_ = 0.02, *p* = 0.98), after-interference (*F*_(2.119)_ = 0.09, *p* = 0.92), and delayed recall (*F*_(2.119)_ = 0.37, *p* = 0.69). RAVLT *post hoc* analyses showed that there was no difference between ε2 carriers and ε3/ε3 individuals (Total recall: *p* = 0.77; bootstrapped BCa 95%CI [−4.25,4.61]; After interference: *p* = 0.84; bootstrapped BCa 95%CI [−1.47,1.43]; Delayed recall: *p* = 0.77; bootstrapped BCa 95%CI [−1.30,1.35]), ε2 carriers and ε4 carriers (Total recall: *p* = 0.98; bootstrapped BCa 95%CI [−4.59,4.07]; After interference: *p* = 0.80; bootstrapped BCa 95%CI [−1.35,1.56]; Delayed recall: *p* = 0.52; bootstrapped BCa 95%CI [−0.99,1.70]), and ε3/ε3 individuals and ε4 carriers (Total recall: *p* = 0.72; bootstrapped BCa 95%CI [−2.92,1.94]; After interference: *p = 0.92*; bootstrapped BCa 95%CI [−0.84,0.98]; Delayed recall: *p* = 0.59; bootstrapped BCa 95%CI [−0.59,1.04]).

Navigation strategy and RAVLT performance were included into a multinomial logistic regression model to assess their association with *APOE* genotype. Sex, age, and education were included in the model as covariates. Assessing the contribution of the predictors, comparing ε2 carriers to ε3/ε3, only navigation strategy had a significant b-coefficient (*b* = 1.34; *OR* = 3.82, *p* = 0.02). Spatial strategy users are significantly more like to be ε2 carriers than ε3 homozygous. The same results were observed when comparing ε2 carriers to ε4 carriers (*b* = 1.44; *OR = 4.2*, *p = 0.03*). Spatial strategy users are more likely to be ε2 carriers than ε4 carriers. The Wald statistic for the b-coefficient of RAVLT total recall (ε2 vs. ε3/ε3: *b* = 0.03, *OR* = 1.03, *p* = 0.66; ε2 vs. ε4: *b* = 0.08, *OR* = 1.08 *p* = 0.27), AI (ε2 vs. ε3/ε3: *b* = 0.03, *OR* = 1.03, *p* = 0.85; ε2 vs. ε4: *b* = 0.07, *OR* = 1.07, *p* = 0.72), and delayed recall (ε2 vs. ε3/ε3: *b* = −0.10, *OR* = 0.90, *p* = 0.65; ε2 vs. ε4: *b* = −0.31, *OR* = 0.74, *p* = 0.21) were not significant. Therefore, navigation strategy has a stronger association with APOE genotype than RAVLT performance.

### APOE, Navigation Strategies, and Hippocampal Gray Matter

**Figure 3:**
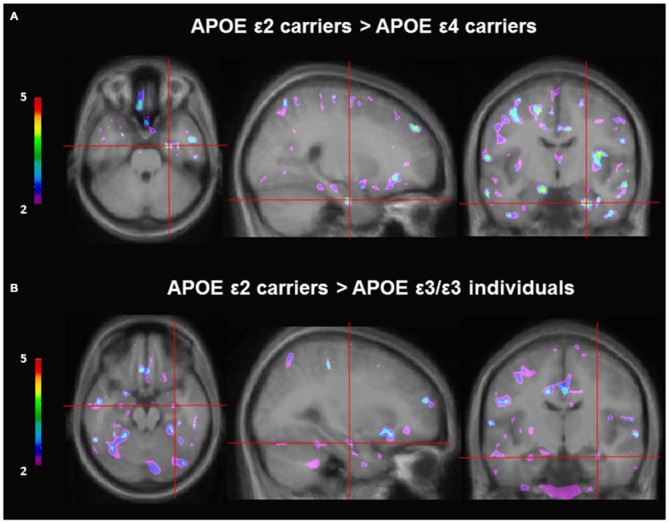
**Gray matter contrast of APOE ε2 carriers and non-ε2 carriers using voxel-based morphometry (VBM). (A)** APOE ε2 carriers (*n* = 5) have more gray matter in the hippocampus compared to APOE ε4 carriers (*n* = 11; *t* = 4.15, *p* < 0.001; *x* = 24, *y* = −10, *z* = −29). **(B)** APOE ε2 carriers (*n* = 5) have more gray matter in the hippocampus compared to APOE ε3 homozygous individuals (*n* = 21; *t* = 3.00, *p* < 0.005; *x* = 30, *y* = −13, *z* = −20).

VBM was used to investigate gray matter differences among the different genotype groups: *ε*2 carriers (*n* = 5), ε3/ε3 (*n* = 22) and ε4 carriers (*n* = 11). Separate analyses were performed with sex, age, and education included as covariates. Given that there is an association between navigation strategy and gray matter in the hippocampus (Bohbot et al., 2007), navigation strategy was included as a covariate in the VBM analyses. As per our hypotheses, we found that ε2 carriers had significantly more gray matter in the right hippocampus than ε4 carriers (*t* = 4.15, *p* < 0.001; *x* = 24, *y* = −10, *z* = −29; Figure 3). These results remained significant when sex, age, education, and navigation strategy were included as covariates (*t* = 4.26, *p* < 0.001; *x* = 24, *y* = −10, *z* = −29). APOE ε2 carriers also had more gray matter in the hippocampus compared to ε3/ε3 individuals (*t* = 3.00, *p* < 0.005; *x* = 30, *y* = −13, *z* = −20). These results remain the same with sex, age, education, and strategy included as covariates (*t* = 3.56, *p* < 0.001; *x* = 30, *y* = −13, *z* = −21). APOE ε2 carriers also had more gray matter in the middle temporal gyrus (*t* = 7.48, *p* < 0.05 bonferroni whole brain corrected; *x* = −44, *y* = −40, *z* = −3) and precuneus (*t* = 7.95, *p* < 0.05 bonferroni whole brain corrected; *x* = 20, *y* = −57, *z* = 43) compared to ε3 homozygous individuals. There was no difference in gray matter in the hippocampus between ε3/ε3 individuals and ε4 carriers.

## Discussion

The current study is the first to show an impact of the *ε*2 allele on cognition in healthy young adults. A significantly higher proportion of *ε*2 allele carriers use spatial strategies compared to *ε*3 homozygous individuals and *ε*4 carriers. In contrast, *ε*3/*ε*3 individuals and *ε*4 allele carriers use response strategies to a greater extent. *ε*2 allele carriers also have more gray matter in the hippocampus compared to *ε*3 homozygous individuals and *ε*4 allele carriers.

In the current study, we demonstrated an association between the *ε*2 allele and increased use of hippocampus-dependent spatial strategies. The advantage of looking at navigation strategies is that they assess differences in the predominant memory system used during navigation rather than cognitive impairment. As such, in past studies, navigation strategies have been sensitive to various genetic and non-genetic factors in young adults (Banner et al., 2011; Bohbot et al., 2011, 2013; Dahmani and Bohbot, 2015; West et al., 2015; Hussain et al., 2016). To date, standard neuropsychological tests have not been able to detect cognitive differences between *ε*2 allele carriers and *ε*3 homozygous individuals and *ε*4 carriers (Alexander et al., 2007; Alexopoulos et al., 2011). A large reason is the scarce body of literature, especially in young adults, that examines the effects of the *ε*2 allele on cognition. Due to the low frequency of this genotype, this group is often excluded from analyses or combined with *ε*3 homozygous individuals to form a non-*ε*4 carrier group. The frequency of the *ε*2 allele is approximately 8%, the *ε*3 allele is 80%, and the *ε*4 allele is 12% (Zannis et al., 1981). Another factor lies in the tests that are used. Tests that assess memory impairments will not be sensitive in young adults, as was demonstrated in the current study using the RAVLT. There was no difference in performance on the standard verbal learning test between the *APOE* groups and the multinomial model demonstrated that navigation strategy is more sensitive to APOE genotype than performance on the RAVLT. Alexopoulos et al. (2011) and Alexander et al. (2007) tested young adults on standard memory and executive function tasks. Both studies compared performance between *ε*2 carrier and *ε*3 homozygous and *ε*4 carrier young adults and found no difference in performance. Therefore, the current study is the first to demonstrate cognitive differences between *ε*2 carriers and non-*ε*2 carriers.

We assessed gray matter in the hippocampus in *ε*2 carriers in comparison to *ε*3 homozygous individuals and *ε*4 carriers. We found that *ε*2 carriers have more gray matter in the hippocampus compared to *ε*3 homozygous individuals and *ε*4 carriers while there was no difference in gray matter in the hippocampus between *ε*3 homozygous individuals and *ε*4 carriers. Given the low frequency of the *ε*2 allele, few studies have looked at the structural differences in young adults between *ε*2 carriers and non-carriers. One study in children demonstrated that *ε*2 carriers have greater cortical thickness in the entorhinal cortex compared to *ε*3 homozygous individuals and *ε*4 carriers (Shaw et al., 2007). Furthermore, in young adults, Alexopoulos et al. (2011) showed that *ε*2 carriers have a larger hippocampal volume compared to *ε*4 carriers. The current results should be interpreted in light of the small sample size limitation of the *ε*2 and *ε*4 groups. However, despite our limited sample size, the current results are consistent with other studies, suggesting that from an early age, there are structural differences in the hippocampus in *ε*2 allele carriers compared to the other genotype groups.

The majority of studies that have looked at the cognitive and structural effects of the *ε*2 allele have been done in older adults. In older adults, the *ε*2 allele is associated with decreased cognitive decline in both healthy individuals (Helkala et al., 1995, 1996; Wilson et al., 2002; Blair et al., 2005) and patients with AD (Serrano-Pozo et al., 2015). Furthermore, the *ε*2 allele is associated with a decreased risk of AD (Corder et al., 1994) and fewer AD-related neuropathology such as Aβ plaques (Chiang et al., 2010; Serrano-Pozo et al., 2015). It is also associated with longevity (Schachter et al., 1994; Blanche et al., 2001; Frisoni et al., 2001) and within centenarians, there is a significantly higher proportion of *ε*2 carriers compared to young adults (Schachter et al., 1994). Structurally, the *ε*2 allele is associated with decreased hippocampal atrophy rates in cognitively healthy individuals (Chiang et al., 2010) and larger hippocampal volumes in patients with dementia (Liu et al., 2010), however there are some inconsistencies in the literature concerning whether older adult *ε*2 carriers have a larger hippocampal volume (Barboriak et al., 2000; Den Heijer et al., 2002). Cortical thickness in the entorhinal cortex has also been shown to be greater in middle-aged *ε*2 carriers (Fennema-Notestine et al., 2011), healthy older adult *ε*2 carriers (Fan et al., 2006), and *ε*2 carriers with mild cognitive impairment and AD (Liu et al., 2010). There is therefore ample evidence to suggest that the *ε*2 allele is protective against cognitive decline and AD in older adults.

The present findings that *ε*2 carriers use hippocampal-dependent spatial strategies to a greater extent and have more gray matter in the hippocampus than the other *APOE* genotype groups may contribute to the explanation of the protective effects of the *ε*2 allele. More gray matter in the hippocampus in *ε*2 carriers may serve to be protective in that these individuals may be able to withstand more cortical atrophy with aging before the manifestation of cognitive impairments. Indeed, lower hippocampal volume is a risk factor for cognitive impairment and AD (Kaye et al., 1997; Jack et al., 1999; Detoledo-Morrell et al., 2004; Stoub et al., 2005; Jagust et al., 2006). Furthermore, the predominant use of hippocampal-dependent spatial strategies during navigation may stimulate the hippocampus to a greater extent than the other genotype groups. In support of this, Iaria et al. (2003) demonstrated in an fMRI study that young adults who use spatial strategies have significantly more fMRI activity in the hippocampus compared to those who use response strategies. This study was done with the same task as was used in the current study. Furthermore in rodents, Lerch et al. (2011) showed that spatial memory training led to increased gray matter in the hippocampus. Preliminary results in humans also support these findings showing that, in older adults, increased use of spatial strategies leads to increased gray matter in the hippocampus (Bohbot et al., 2015). Although in the current study causality cannot be determined, a larger hippocampus may lead to increased use of spatial strategies and this in turn may continue to stimulate the hippocampus and serve as a protective mechanism with aging. However, this will have to be further investigated with an fMRI longitudinal study.

Much of the research on the *APOE* gene has been focused on the *ε*4 allele. The *ε*4 allele is the strongest known genetic risk factor for AD (Corder et al., 1993) and is more frequent in the population than the *ε*2 allele. In young adults, there are some inconsistencies regarding the effects of the *ε*4 allele on cognition and brain structure and function. Some studies have found that the *ε*4 allele is associated with better cognitive performance (Han et al., 2007; Mondadori et al., 2007) while others have found no differences in cognitive performance (Liu et al., 2010; Bunce et al., 2011; Richter-Schmidinger et al., 2011). Similarly, some studies have found that *ε*4 allele carriers have a smaller hippocampal volume (O’Dwyer et al., 2012) while others have failed to find this effect (Mondadori et al., 2007; Richter-Schmidinger et al., 2011). Brain function has also been inconsistent within this group, with some studies showing that *ε*4 allele have more fMRI activity in the hippocampus (Filbey et al., 2006; Filippini et al., 2009; Dennis et al., 2010) while others show the opposite effect (Mondadori et al., 2007). Given the inconsistencies in the literature it is difficult to determine the exact nature of the effect of the *ε*4 allele in young adults. Many of the inconsistencies may arise from differences in the behavioral and fMRI tasks used and methods to measure hippocampal volume. Variable sensitivity of measures may be the underlying reason why it is difficult to determine the effects of the *ε*4 allele in young adults. For example, under conditions of constant start and target positions, the response strategy is more efficient than the spatial strategy (Iaria et al., 2003). On the other hand, with variable start and target positions, the spatial strategy is more efficient (Etchamendy and Bohbot, 2007). As such, discrepant results in the literature would result from the sensitivity of the variables measured. For this reason, it is not surprising that in the current study we did not observe a difference between *ε*3 homozygous individuals and *ε*4 carriers. It is possible that the effects of the *ε*4 allele are more pronounced with aging, when compensatory strategies are less effective, as opposed to young adults where these effects are more difficult to detect. In support of this, in older adults, the effects of the *ε*4 allele are more consistent with most studies finding that the *ε*4 allele is associated with poor cognitive performance (Schmidt et al., 1996; Reiman et al., 1998; Driscoll et al., 2005; Lemaitre et al., 2005; Berteau-Pavy et al., 2007; Liu et al., 2010), decreased hippocampal volume (Reiman et al., 1998; Den Heijer et al., 2002; Lemaitre et al., 2005; Cherbuin et al., 2007; Crivello et al., 2010), decreased cortical thickness in the entorhinal cortex (Burggren et al., 2008), and decreased hippocampal function (Filippini et al., 2011). However, even within older adults, there are some discrepancies between studies (Trachtenberg et al., 2012).

The current study should be viewed in the light of certain limitations. Studies examining the effects of the *ε*2 allele often suffer from sample size limitations due to the low frequency of the genotype in the general population. Similarly, in the current study, the MRI sample was relatively small and therefore should be aimed to be replicated in a larger cohort. However, despite the low sample size, the current MRI findings are consistent with others in the literature. It will also need to be confirmed with an fMRI study that *ε*2 carriers indeed have more fMRI activity in the hippocampus. Based on our previous research in young adults which showed that spatial strategy users have more fMRI activity in the hippocampus compared to response strategy users and given our current findings that *ε*2 carriers use spatial strategies to a greater extent, we hypothesize that *ε*2 carriers will indeed have more fMRI activity in the hippocampus. One inconsistency that we found was that despite a higher proportion of *ε*2 carriers using a spatial strategy, there was no difference in probe scores between the three genotype groups. The probe trial is used to perform an overall validation of the strategy assessments obtained from the verbal reports. In past studies, the probe trial has been used to confirm that spatial strategy users rely on landmarks to a greater extent (Iaria et al., 2003; Bohbot et al., 2007; Schwabe et al., 2012; West et al., 2015). As such, probe errors are entirely related to navigation strategy and indeed, in the current study, spatial strategy users made more errors on the probe trial than response strategy users. Among the *APOE* groups, both the *ε*2 carriers and non-carriers used a mix of spatial and response strategies (*ε*2 carriers: 61% and 39% respectively). Therefore, it is not surprising that there was no significant performance difference on the probe trial between the *APOE* groups. In other words, differences in probe scores, i.e., when landmarks are removed from the virtual environment, reflect the lack of use of landmarks, not genotype. There is also evidence that hormonal status can also influence navigation strategies. High estrogen or progesterone levels in female rats are associated with increased volume in the hippocampus (Protopopescu et al., 2008; Qiu et al., 2013) and increased use of spatial strategies (Korol et al., 2004; Hussain et al., 2016). In the current study, we did not examine hormonal status or menstrual cycle phase; however, in future studies it would be of interest to look at these variables in relation to navigation strategies.

In conclusion, this is the first study to show the beneficial effects of the APOE ε2 allele on hippocampus-dependent cognition in healthy young adults. Young adult *ε*2 carriers used spatial strategies to a greater extent than *ε*3 homozygous individuals and *ε*4 carriers. Furthermore, young adult *ε*2 carriers had more gray matter in the hippocampus compared to *ε*3 homozygous individuals and *ε*4 carriers. Based on the association between the ε2 allele and increased use of hippocampus-dependent spatial strategies, the study suggests an important potential mechanism by which the APOE ε2 allele is associated with decreased cognitive decline and risk of developing AD. Since spatial strategies have been shown to be effective at promoting growth in the hippocampus (Lerch et al., 2011; Bohbot et al., 2015), early intervention trials focusing on spatial memory may provide a novel opportunity for clinical trials aimed at decreasing conversion rates to AD.

## Author Contributions

KK contributed to the genetic, cognitive, and MRI analyses and wrote the manuscript. VB contributed to the data collection, the genetic and cognitive analyses and the writing of the manuscript. HB collected a portion of the MRI scans, and genetic analyses. JP contributed to the theoretical and editorial aspects of the manuscript. RJ contributed to the genetic analyses and editorial aspects. VDB contributed to the conceptual and experimental design, behavioral and MRI data collection, all the theoretical analyses, and writing of the cognitive neuroscience and MRI aspects of this manuscript.

## Conflict of Interest Statement

The authors declare that the research was conducted in the absence of any commercial or financial relationships that could be construed as a potential conflict of interest.
